# Supplementary material for: Wood formation of drought-resistant Eucalyptus cladocalyx under cyclical drought treatment
Source: Quant Plant Biol. 2025 Apr 7;6:e12. doi: 10.1017/qpb.2025.7 (PMC12035783; doi:10.1017/qpb.2025.7)
Supplement: Gama et al. supplementary material [file S2632882825000074sup001.docx]

**Part 1: Supplementary data**

**Ambient climate data during the study period**

Table S1: La Colline, Stellenbosch rainfall total per month(mm) for the year 2017 during experimental trial

| **Month** | **Monthly rainfall(mm)** |
| --- | --- |
| January | 13.5 |
| February | 0.0 |
| March | 7.7 |
| April | 33.8 |
| May | 10.5 |
| June | 142.9 |

Table S2: statistical values of measured anatomical properties throughout the experimental trail

| **Cell property** | **Treatment** | **Block** | **Mean** | **Standard deviations** |
| --- | --- | --- | --- | --- |
| Vessel area(um^2^) | Periodic irrigation | A | 1183.49 | 728.34 |
|  |  | D | 1375.01 | 669.41 |
|  | Continuous irrigation | B | 1438.40 | 798.52 |
|  |  | C | 1207.01 | 695.17 |
| Vessel frequency(mm^2^) | Periodic irrigation | A  D | 141.43  98.24 | 50.59  22.61 |
|  | Continuous irrigation | B | 106.17 | 36.25 |
|  |  | C | 101.16 | 42.61 |
| Fiber area(um^2^) | Periodic irrigation | A  D | 288.51  257.93 | 105.34  100.29 |
|  | Continuous irrigation | B | 261.86 | 94.37 |
|  |  | C | 257.85 | 82.38 |
| Fiber thickness(um) | Periodic irrigation | A  D | 4.35  4.12 | 1.00  0.94 |
|  |  | B | 4.35 | 1.12 |
|  | Continuous irrigation | C | 4.133 | 0.86 |

**Part 2: Image processing and calculations**

**Methods**

**Required Microscopic data**

Images acquired from microscopic sectioning techniques can be analysed with this technique. The format of the images should be a tiff file and/or a file supported by Qupath. The images should be brightfield images and of clear and good quality.

**Software equipment**

Qupath and R can be run on Windows XP, Mac OSX and Linux based on 32 and 64-bit operating systems. Sufficient RAM is needed to be able to store the images.

**Software**

- QuPath: <https://qupath.github.io/>
- Install R: <https://cran.rstudio.com/> and
- R studio: <https://www.rstudio.com/products/rstudio/download/>

**Scripts to download**

**Classification and image extraction (Keret et al.,2024):**

https://doi.org/10.5281/zenodo.8006449,https://doi.org/10.5281/zenodo.8006637, https://doi.org/10.5281/zenodo.8006687, https://doi.org/10.5281/zenodo.8006705.

**Functional traits calculations:**

Fiber, Vessel, and Cell Dynamics scripts:

http://doi.org/ [10.5281/zenodo.13799117]

**Installation of Qupath**

1. Download and install QuPath (0.4.3 version) from <https://qupath.github.io/>
2. Set up Qupath-0.4.3 wizard.
3. Complete set up by clicking Finish.

**Sample preparation**

The brightfield images of *Eucalyptus grandis* section were acquired using Nikon Eclipse E400 Microscope (Nikon Instruments, Melville, New York, USA) using 20X imaging objective for microscopic examination of the differentiating xylem. The protocol of Keret et al., 2024 was followed for tissue processing, embedding, and sectioning of samples stems sample.

**Acquiring of x and y coordinates**

Qupath was used to obtain x and y coordinates of the cambium and vessels as well as other anatomical elements. The images were added and displayed under the project. The detection script was used to make cell detections of microscopic images. To get the detection script running, click on Automate tab on Qupath and hereafter on show script editor tab to upload Cell_detection script. Ensure to change the last line of the code, changing the path of the script so that Qupath recognises where the classifier is located. To run the script, select Run for project and selected all the images under the project. Cell detection results can be obtained using the Extraction script which needs to be uploaded via script editor. The Extraction script will save all the detected and measured cell variables. In these variables, centroid points (x and y coordinates) of all the cells will be included. In this paper, we will use the coordinates of the cambium and xylem. Alternatively, the x and y coordinates of the cambium and vessels can also produce by other software techniques such as Image J or Bitplane Imaris using the specified images.

**Detailed steps of the image analysis process**

Follow option A to detect and classify cells on Qupath; follow option B to analyse the measured data on R and follow option C to add environmental data and allocate different growth zone and create figures.

**A.** **Detection and classifying of multicellular images on Qupath.**

1. *Software installation.* Download and install QuPath (0.4.3 version) from <https://qupath.github.io/>
2. *Creating project.* Open the software and navigate to “File” / “Project”/ “Create project”, then select the empty folder you want to create project in (can create directory).
3. *Adding images.* To add images to the project, navigate to “Project” icon then press on “Add images”/ “Set image type”: “Other”/” Choose files” to add the images and press “Import”. Multiple images can be added at the same time. The list of images will then show under the project file. When the images have been added, they will show under the “Image list” which is within the “Project” icon. The project file will store all information related to the images.
4. *Adding Cell detection.* Download Cell_detection script, Classifier and Extraction script and save under project folder: To run script, navigate to “Automate”/ “Show script editor” on Qupath. Hereafter navigate to “File”, firstly add in “Cell_detection script” and “Open” script in the folder it is saved in.
5. *Changing line of code*. When the “Cell_detection script” is now open, change last line of code: “RunObjectClassifier”, change path to lead to the classifier. This can be simply done by copying the path of the classifier and codes ends with Classifier.json. Change the direction of the slashes to forward slash for the script to run.
6. *Changing image size.* Adjust the image width and height according to your specific image size.

*Running cell detection*. Navigate to “Run”/ “Run for project” and select all images to be added then press “OK”.

1. *Extracting cellular data.* Detection results can be extracted using “Extraction script”, “File”, firstly add in “Extraction script” and “Open” script in the folder it is saved in and run for project and save images as a single appended csv file.

**B. Analysing measured anatomical variables on R.**

1. *Installation of R.* To analyse the measured variables, this must be done on R. Firstly, download and Install R: <https://cran.rstudio.com/> as well as R studio: <https://www.rstudio.com/products/rstudio/download/>
2. *Creating R project.* Open R and Navigate to “File”, “New Project” and name the project and select directory. Ensure to always work in the directory you have chosen. Inside this directory, create folder in that directory. One folder should be named “Data” where it will keep all the raw data, and the second folder will be name “ouput”. This folder will keep the created plots.
3. *Saving files to directory.* Download the l zoning _script” and ensure this script is saved in the folder ouput_zoning folder and both this folder and the project are under the same directory. The appended csv file from part A (vi) should be saved under the data folder in the same directory as the project.
4. *Importing image data*. The downloaded csv file from Qupath file is used in the current format it is in. Hereafter the zoning _script” needs to be opened. After opening script, start running the script from the line that says, “load packages”, run both these lines of codes and ensure data shows on the R environment on the side. The csv file can then be imported to R following the line code “import data”. User needs to change the code in accordance with their directory and ensure name of the file is correct and its the same as the one that appears in the directory. If the file name is not the same, the data will not load or if a different directory is used, this will also cause the script not to work. Ensure name of the file s correct and there are spaces within the name.


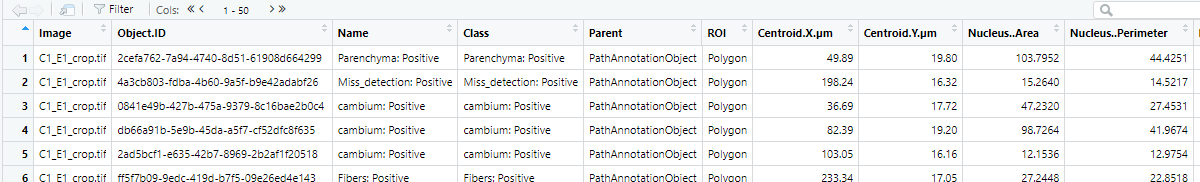
Table 1: Table showing example of data uploaded on R environment from appended file.

1. *Running script.* Continue to run line of code line by line to see the resulting tables and plots created. The first plot under line code will illustrate the structure of the cambium cells and hereafter the script in line code will also create a best line of fit through the cambium. The second plot in line code will add in vessel data and this plot will show the cambium and vessel structure for each image.

1. *Finding Cy*. In the last part of the cambial zoning script will create an equation that will find the y position of the vessels that can be used to determine their position within the cambium and how far they are with different growth points.

**C. Adding Environmental data.**

*Creating diameter file*. To add environmental data to the script, the data first needs to be organised in this manner; create csv file containing date and diameter measurements with columns names:

- Date(year/month/day)
- Diameter (measurements taken during the trial)
- Tree_id (identification of each tree)
- Treatment (use Cl if trees were continuously irrigated or PI if trees were periodically irrigated)
- Day_no (includes the dates of the experiment)
- Cycle (includes the time periods allocated to the experiment)

Once columns have been created, file needs to be saved as Diameter_data and saved under the Data folder, which is in the same directory as the project.

Table 2: Table showing an example of environmental data and information to include.

| Date | Diameter | Tree_id | Treatment | Day_no | Cycle |
| --- | --- | --- | --- | --- | --- |
| 2021/04/23 | 3.96 | C1 | Cl | 0 | T1 |
| 2021/04/23 | 3.47 | C2 | Cl | 0 | T2 |
| 2021/04/23 | 3.61 | C3 | Cl | 0 | T3 |

1. *Import diameter data.* Using the same zone script under line code ensure that the directory is the same as where the script is located. Ensure that the name of the file is the same as the name written on the line of code.to R under line code . Hereafter run the line of code to import the growth data and ensure that it appears on within the R environment. Run code from line to line. This will result in creating change in diameter as well as creating growth zones, this will be created for all the images. The final output will create a plot illustrating the distance of vessels from the cambium and as well as how many vessels and fibres were created per zone.
2. For statistical analysis use the Fiber, Vessel, and Cell Dynamics scripts. Each script began by loading and preprocessing data, including temperature, humidity, diameter, leaf water potential, wood anatomy, and cambial activity

**
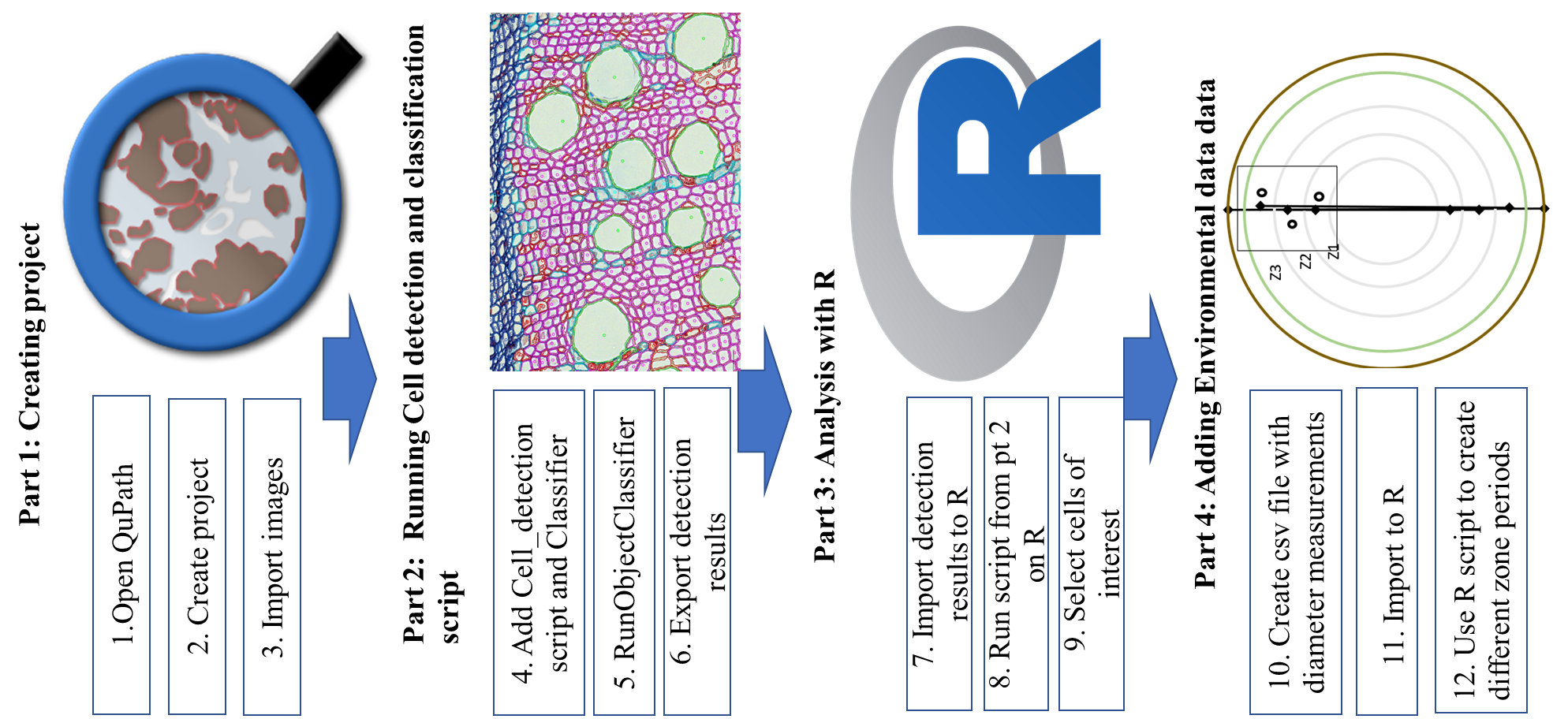
**

Figure 1: Summary of steps for analysing, detecting, and classifying microscopic cells.
